# Training in communication addressed to doctors and patients in the HIV/AIDS area

**DOI:** 10.1186/1471-2334-13-S1-P24

**Published:** 2013-12-16

**Authors:** Mioara Predescu

**Affiliations:** 1 Romanian HIV/AIDS Center, National Institute for Infectious Diseases “Prof. Dr. Matei Balş”, Bucharest, Romania

## Background

The Romanian HIV/AIDS Center in the National Institute for Infectious Diseases “Prof. Dr. Matei Balş” in partnership with the Romanian Association against AIDS (ARAS) and Data Center Solutions implemented a project destined to improve communication between doctors and HIV patients.

## Methods

The project aimed to experiment a new type of training by communication, namely online communication through an electronic platform. The training focused on two different approaches: an online, real-time class designed for patients, taught by physicians, psychologists and social workers from the National Institute for Infectious Diseases “Prof. Dr. Matei Balş” and another class addressed to physicians. The latter was uploaded on the website so that it could be accessed by doctors, depending on their spare time and interest in a certain topic.

## Results

The classes for physicians were held by teams of doctors, psychologists and social workers from the National Institute for Infectious Diseases “Prof. Dr. Matei Balş”. More valid data on the rate of access are to be released by specialists in this type of communication from Data Center Solutions.

The classes for patients were deployed throughout 10 online sessions, twice a week and were held by teams made of doctors, psychologists and social workers from the National Institute for Infectious Diseases “Prof. Dr. Matei Balş”. They consisted of an initial segment of teaching, kept by the teaching teams and a second segment of questions and answers formulated by patients. ARAS was responsible with mobilizing and informing those interested in the subject, so that anyone who owed a PC at their personal residence could access the class, while other interested patients were gathered in ARAS centers all around the country.

## Conclusion

This new method of communication has had, so far, a great popularity, this representing the underlying reason that the initial model of training in 2012 was resumed, with added modifications suggested by participants’ questions that targeted especially injecting drug users. The largest interest revolved around treatment and prevention. The innovative communication method was unanimously accepted as highly useful. Recording of presentations were later on resumed in schools and some penitentiaries in Romania. The classes’ recordings were also displayed in schools and penitentiaries, with approximately 180 hours of replay and a mean share of participation of 8000 viewers. They had the possibility to listen to questions and answers formulated during the initial class to which they added their own questions addressed to ARAS representatives.

